# Study of the inflammatory activating process in the early stage of *Fusobacterium nucleatum* infected PDLSCs

**DOI:** 10.1038/s41368-022-00213-0

**Published:** 2023-02-08

**Authors:** Yushang Wang, Lihua Wang, Tianyong Sun, Song Shen, Zixuan Li, Xiaomei Ma, Xiufeng Gu, Xiumei Zhang, Ai Peng, Xin Xu, Qiang Feng

**Affiliations:** 1 Department of Implantology, School and Hospital of Stomatology, Cheeloo College of Medicine, Shandong University & Shandong Key Laboratory of Oral Tissue Regeneration & Shandong Engineering Laboratory for Dental Materials and Oral Tissue Regeneration, Jinan, China; 2 Department of Human Microbiome, School and Hospital of Stomatology, Cheeloo College of Medicine, Shandong University & Shandong Key Laboratory of Oral Tissue Regeneration & Shandong Engineering Laboratory for Dental Materials and Oral Tissue Regeneration, Jinan, China; 3 Department of Orthodontics, School and Hospital of Stomatology, Cheeloo College of Medicine, Shandong University & Shandong Key Laboratory of Oral Tissue Regeneration & Shandong Engineering Laboratory for Dental Materials and Oral Tissue Regeneration, Jinan, China; 4 State key laboratory of microbial technology, Shandong University, Qingdao, China

**Keywords:** Cellular microbiology, Gene regulatory networks

## Abstract

*Fusobacterium nucleatum* (*F. nucleatum*) is an early pathogenic colonizer in periodontitis, but the host response to infection with this pathogen remains unclear. In this study, we built an *F. nucleatum* infectious model with human periodontal ligament stem cells (PDLSCs) and showed that *F. nucleatum* could inhibit proliferation, and facilitate apoptosis, ferroptosis, and inflammatory cytokine production in a dose-dependent manner. The *F. nucleatum* adhesin FadA acted as a proinflammatory virulence factor and increased the expression of interleukin(IL)-1β, IL-6 and IL-8. Further study showed that FadA could bind with PEBP1 to activate the Raf1-MAPK and IKK-NF-κB signaling pathways. Time-course RNA-sequencing analyses showed the cascade of gene activation process in PDLSCs with increasing durations of *F. nucleatum* infection. NFκB1 and NFκB2 upregulated after 3 h of *F. nucleatum*-infection, and the inflammatory-related genes in the NF-κB signaling pathway were serially elevated with time. Using computational drug repositioning analysis, we predicted and validated that two potential drugs (piperlongumine and fisetin) could attenuate the negative effects of *F. nucleatum*-infection. Collectively, this study unveils the potential pathogenic mechanisms of *F. nucleatum* and the host inflammatory response at the early stage of *F. nucleatum* infection.

## Introduction

Periodontitis is a widespread chronic immunoinflammatory disease of periodontal tissues, that affects more than 60% of the global adult population.^1,2^ The pathogenesis of periodontitis is convoluted, which involves microbial challenges, host genetic variations, and acquired environmental stressors.^3,4^ Among the numerous risk factors, the increase in pathogenic microbes in the subgingival plaque is widely accepted as a necessary prerequisite for the development of periodontitis. *Fusobacterium nucleatum* (*F. nucleatum*) is one of the most frequently detected pathogens and has attracted increasing attention in recent years as an opportunistic pathogen in many systematic diseases, such as colorectal cancer,^5^ cardiovascular diseases,^6^ Alzheimer’s disease,^7^ and adverse pregnancy outcomes.^8^

*F. nucleatum* is an invasive bacterium that can induce a variety of host responses.^9^ Clinical studies have shown that the prevalence of *F. nucleatum* increases with the severity and progression of periodontitis.^10,11^ *F. nucleatum* can invade various host cells, such as epithelial and endothelial cells, monocytes and fibroblasts, to initiate a cascade of inflammation and induce the secretion of the proinflammatory chemokines interleukin(IL)-6 and IL-8.^12,13^ Toxic proteins are an important way for bacteria to exert pathogenicity, and *F. nucleatum* expresses a variety of virulence factors to induce various host responses.^14^ For instance, RadD and Fap2 induce lymphocyte apoptosis,^15^ and FadA mediates host-cell binding and invasion in epithelial cells.^16,17^ This evidence indicates that *F. nucleatum* might have different pathogenic mechanisms to exert its pathogenic effect on different cell types.

As a main cell type in the periodontal ligament, periodontal ligament stem cells (PDLSCs) play an indispensable role in maintaining periodontal homeostasis.^18^ According to emerging evidence, the inflammatory environment caused by periodontitis leads to dysfunction and pyroptosis in PDLSCs.^19^ Zhao et al. demonstrated that treatment with butyrate, a secondary metabolite of periodontal pathogens, could induce ferroptosis in periodontal ligament fibroblasts and regulate cell survival and death.^20^ However, the biological characteristics and changes in gene regulation in PDLSCs caused by *F. nucleatum* have not yet been fully clarified.

In this study, we explored the pathogenic effects of *F. nucleatum* and the host response of PDLSCs in the early stage of infection. We evaluated the changes in the biological activities in PDLSCs during *F. nucleatum* infection and examined the virulent effect of the *F. nucleatum* adhesin FadA. Time-course gene expression analysis was used to reveal gene regulation in response to *F. nucleatum* infection. Finally, coexpression-based computational drug repositioning was used to identify drug candidates to attenuate the pathogenic effects of *F. nucleatum* on PDLSCs.

## Results

Human PDLSCs from healthy and young volunteers were successfully isolated and cultured as described in the Materials and Methods. The cultured PDLSCs exhibited a spindle-shaped fibroblast-like morphology (Fig. S1a). In the multidifferentiation assay, Alizarin Red–positive mineralized matrix and Oil Red O–positive lipid droplets were observed (Fig. S1b, c). Immunophenotypic analysis showed that PDLSCs expressed MSC-specific surface markers, but not hematopoietic or endothelial cell-specific markers (Fig. S1d).

### *F. nucleatum* inhibits proliferation, and facilitates apoptosis, ferroptosis, and inflammatory cytokine production in PDLSCs

**Fig. 1:**
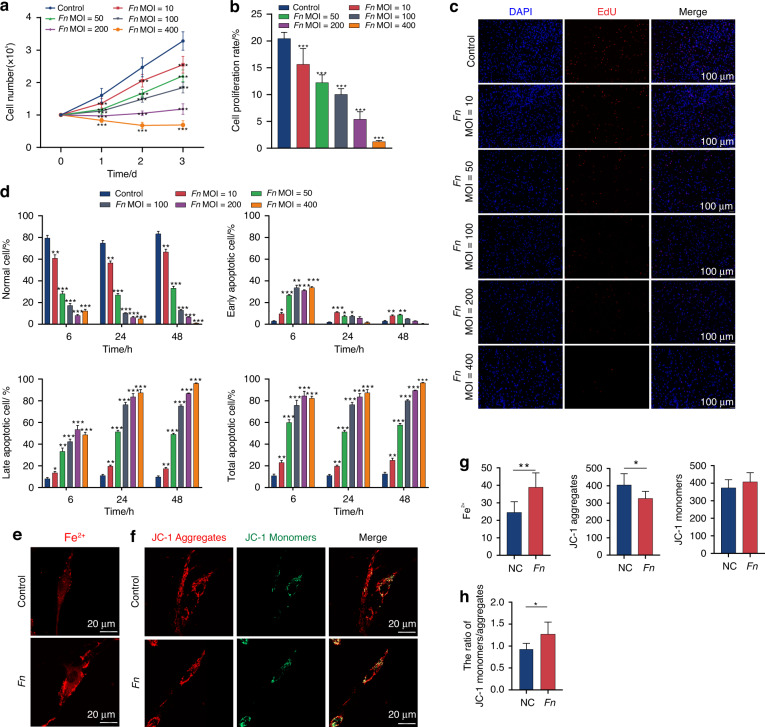
*F. nucleatum* inhibits proliferation, facilitates apoptosis, and ferroptosis in PDLSCs. **a** Cell-counting assay of PDLSCs cocultured with or without *F. nucleatum*. **b**, **c** Cell proliferation rate of PDLSCs detected by EdU assay. Scale bar: 100 μm. **d** Cell apoptosis examined using Annexin V/PI staining. Annexin V-/PI- represents live cells, Annexin V + /PI- early apoptosis, Annexin V + /PI + late apoptosis, and Annexin V-/PI + necrosis. **e** Intracellular Fe^2+^ detected by FerroOrange. Scale bar: 20 μm. **f** Confocal images of JC-1 in PDLSCs. Scale bar: 20 μm. **g** Quantitative assessment of FerroOrange and JC-1 fluorescence. **h** The ratio of JC-1 monomers/aggregates. Data were expressed as mean ± SD. (*n* = 3) (*P < 0.05; **P < 0.01; ***P < 0.001, compared with the control group)

To determine the pathogenic effect of *F. nucleatum* on PDLSCs, we first evaluated the viability of PDLSCs exposed to different MOIs of *F. nucleatum*. The results showed that *F. nucleatum* significantly inhibited the proliferation of PDLSCs in a time- and dose-dependent manner (*P* < 0.001) (Fig. 1a–c). Proliferation was almost blocked at MOIs of 200 and 400. Next, we evaluated the apoptosis ratios of *F. nucleatum*-infected PDLSCs. As shown in Fig. 1d and Fig. S2, *F. nucleatum* significantly increased the apoptosis rate of PDLSCs in a dose- and time-dependent manner (*P* < 0.05). Notably, early apoptosis mainly occurred at 6 h, while late apoptotic cells accounted for a substantial portion of total apoptotic cells at 24 h and 48 h.

Ferroptosis, which is a novel necrotic cell death pathway, is triggered by iron overload.^21^ Perturbations in iron homeostasis are major pathogenic strategies for bacterial infection.^22^ To investigate whether *F. nucleatum* induced ferroptosis in PDLSCs, we compared intracellular free iron levels between normal and *F. nucleatum* infected PDLSCs. The fluorescence intensity of Fe^2+^ was significantly enhanced in the *F. nucleatum*-infected group (*P* < 0.01) (Fig. 1e, g). Iron overload leads to mitochondrial dysfunction, which mainly manifests as mitochondrial membrane potential (MitoMP) depolarization.^23,24^ Therefore, we evaluated the intracellular MitoMP of PDLSCs using JC-1 fluorescent dye and observed that *F. nucleatum* treatment reduced the fluorescence intensity of JC-1 aggregates and enhanced the green fluorescence of JC-1 monomers (Fig. 1f-g). Quantitative analysis showed that the JC-1 monomer/aggregate intensity ratio was increased after *F. nucleatum* infection, indicating that *F. nucleatum*-induced iron overload may impair mitochondrial function in PDLSCs (Fig. 1h). These results first showed that *F. nucleatum* treatment could increase the intracellular labile iron levels and promote MitoMP depolarization in host cells.

**Fig. 2:**
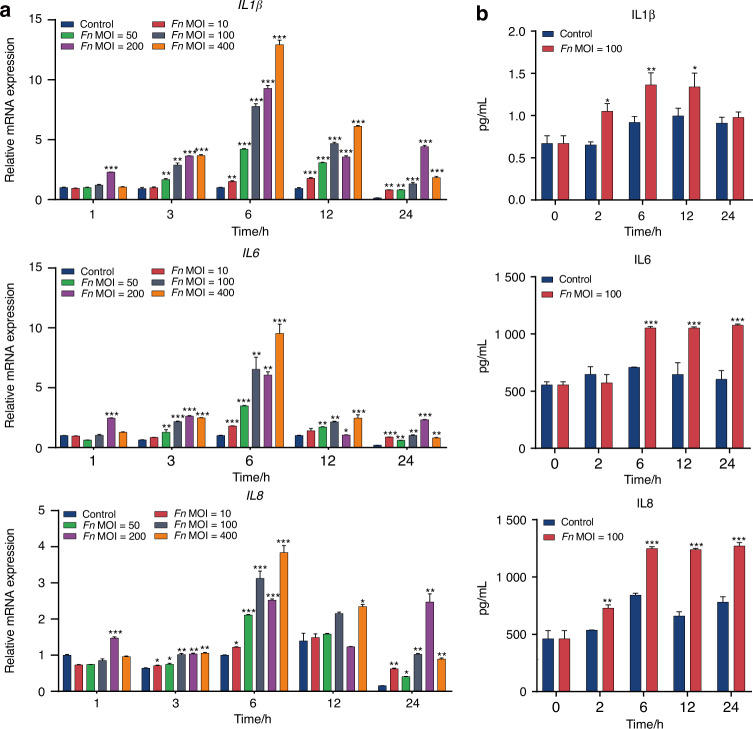
*F. nucleatum* promotes the production of inflammatory cytokines in PDLSCs. **a** The mRNA expression detected by qRT-PCR. **b** Protein levels measured by ELISA. Data were expressed as mean ± SD. (*n* = 3) (*P < 0.05; **P < 0.01; ***P < 0.001, compared with the control group)

We next evaluated whether *F. nucleatum* could trigger inflammatory responses in PDLSCs by qRT-PCR and ELISA. As shown in Fig. 2a, the gene expression levels of *IL-1β, IL-6,* and *IL-8* increased with increasing stimulation time and peaked at 6 h in a dose-dependent manner, and they regressed with the duration of stimulation. At the protein level, *IL-1β, IL-6,* and *IL-8* were also consistently increased with increasing stimulation time in the early stage and reached a maximum level at 12 h (Fig. 2b). These results suggest the potential immunomodulatory effect of PDLSCs under the stimulation of periodontal pathogens.

### FadA activates NF-κB and MAPK signaling pathways by interacting with PEBP1

**Fig. 3:**
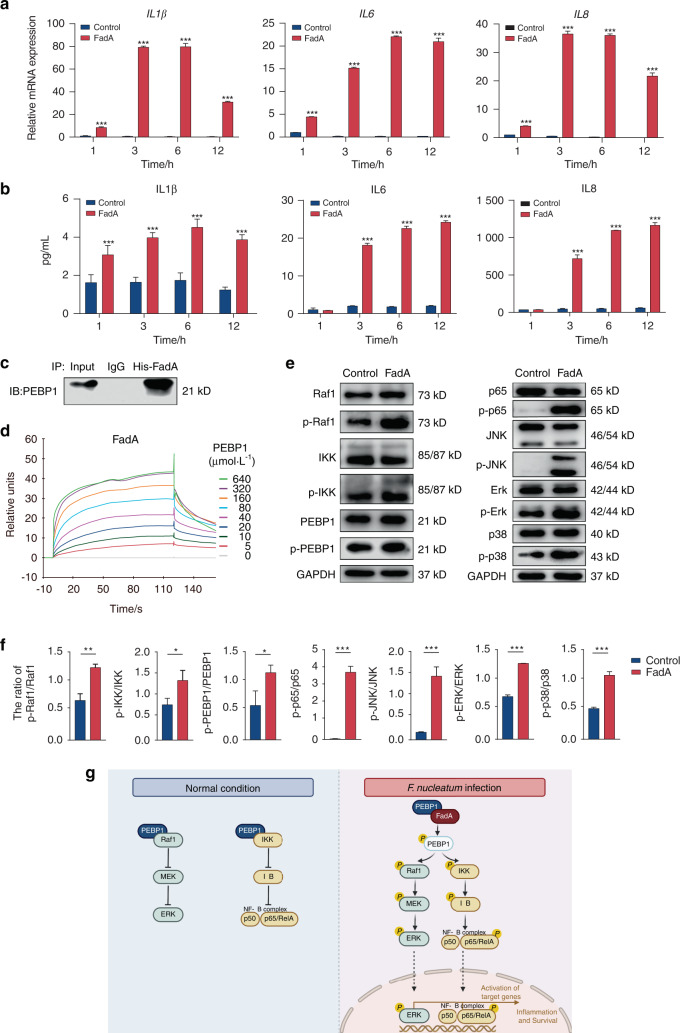
*F. nucleatum* adhesin FadA promotes inflammatory response via interaction with PEBP1. **a** The mRNA expression detected by qRT-PCR. **b** Protein levels measured by ELISA. **c** Co-IP analysis showed the interaction between FadA and PEBP1. **d** SPR analysis of PEBP1 binding to FadA. PEBP1 at various concentrations was used as analyte to detect binding to FadA immobilized on the sensor chip. **e** Effects of FadA on the phosphorylation of Raf1, IKK, PEBP1, p38, JNK, ERK, and NF-κB p65 detected by Western blot analysis. **f** Quantitative analysis of altered protein expression of these proteins used ImageJ. **g** Schematic diagram of the FadA-PEBP1-Raf1-MAPK pathway and FadA-PEBP1-IKK-NF-κB pathway. Created with BiorRender.com. Data were expressed as mean ± SD (*n* = 3) (*P < 0.05; **P < 0.01; ***P < 0.001, compared with the control group)

*Fusobacterium* adhesin A (FadA) has been reported to be one of the most important adhesins and virulence factors of *F. nucleatum*.^16^ To explore the molecular mechanism of *F. nucleatum* infection, we investigated the pathogenic effect of FadA on PDLSCs. We obtained recombinant histidine (His)-tagged FadA through an *E. coli* expression system (Fig. S3a–b). After the addition of 0.5 mg·mL^−1^ FadA protein, the mRNA levels of *IL1β*, *IL6,* and *IL8* were significantly increased compared with the controls (*P* < 0.001) (Fig. 3a). At the protein level, the IL1β level increased at 1 h, while IL6 and IL8 increased at 3 h after FadA stimulation (Fig. 3b).

Next, we isolated FadA-binding proteins by a His pull-down assay, and identified all pull-down proteins by mass spectrometry. Among the candidates (Table S3), a cytoplasmic protein phosphatidylethanolamine-binding protein 1 (PEBP1), which is also known as Raf1 kinase inhibitory protein (RKIP),^25–27^ was proven to be co-immunoprecipitated with FadA by Co-IP assay (Fig. 3c). The direct binding of PEBP1 to FadA was further confirmed by surface plasmon resonance (SPR) analysis (Fig. S3c, d and Fig. 3d).

To study whether FadA induces the inflammatory response by interacting with PEBP1, we first evaluated the phosphorylation state of PEBP1. Figure 3e shows the binding of FadA-PEBP1 phosphorylated PEBP1 at S153. As the devitalization of PEBP1 could activate Raf1 and IKK, we hypothesized that FadA promoted the production of proinflammatory cytokines by activating the NF-κB and MAPK signaling pathways by binding to PEBP1. Western blot analysis showed that Raf1 and IKK were both significantly activated, and ERK-JNK-p38 MAPKs and NF-κB-p65 were subsequently significantly activated in PDLSCs (*P* < 0.05) (Fig. 3e–g). These findings suggest that FadA acts as a pathogenic effector of *F. nucleatum* and can initiate intracellular immune signal transduction in PDLSCs.

### *F. nucleatum* infection induces dynamic gene activation in PDLSCs

**Fig. 4:**
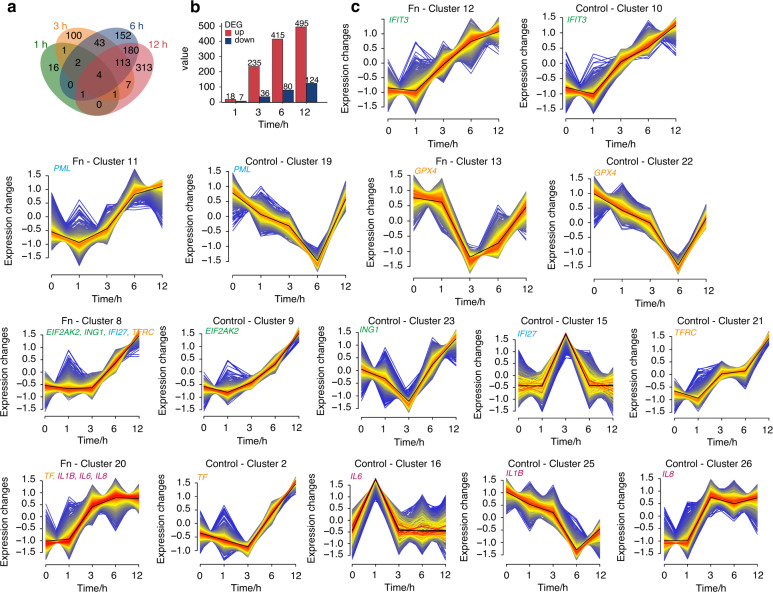
Dynamic gene expression patterns of PDLSCs under F. nucleatum Infection. **a** Venn diagram depicted the extent of overlap between DEGs at different time points in PDLSCs. **b** The histograms of upregulated (red) and downregulated (blue) DEGs at each time point. **c** Mfuzz analysis clustered genes into different classes. Green font represents proliferation-related genes, blue font apoptosis-related genes, orange font ferroptosis-related genes, and red font inflammation-related genes

At present, the gene regulation process in oral cells in the early stage of *F. nucleatum* infection is unknown. We performed time course RNA-seq analysis of PDLSCs under *F. nucleatum* infection for 1 h, 3 h, 6 h, and 12 h. Principal component analysis (PCA) showed that the transcriptomes of the control groups exhibited a stable gene expression state, while the *F. nucleatum*-infected groups changed continuously in a particular pattern (Fig. S4). The gene expression profiles of the control group and experimental group at 1, 3, 6, and 12 h were compared by DESeq2, and 25, 271, 495, and 619 differentially expressed genes (DEGs) were identified at each time point, respectively (Fig. 4a). Among these genes, 18, 235, 415, and 495 were upregulated, while 7, 36, 80, and 124 were downregulated at each time point (Fig. 4b). Notably, the Venn diagram showed that 4 DEGs were consistently upregulated in the *F. nucleatum-*stimulated group across the four time points (Fig. 4a and Fig. S5). *CXCL1* and *CXCL2* are two vital neutrophil chemoattractants. The continuous upregulation of these two chemokines indicated the key role of PDLSCs in recruiting immune cells during *F. nucleatum* infection.

To reveal the gene expression patterns related to the cytological phenotypes that are altered by *F. nucleatum*, we clustered all the genes into 30 expression patterns in control group and 20 clusters in *F. nucleatum* group by Mfuzz analysis (Fig. S6). As shown in Fig. 4c, we found several proliferation-inhibition genes that showed a continuous upregulation trend after *F. nucleatum* infection, including *IFIT3, ING1*, and *EIF2AK2*. Apoptosis-related gene such as *IFI27* and *PML* showed a consistent increasing trend. Notably, some genes related to iron metabolism (such as *TFRC* and *TF*) were continuously upregulated while those associated with ROS detoxification (such as *GPX4*) were gradually decreasing, suggest that *F. nucleatum* could induce ferroptosis by aggravating intracellular iron overload and inhibiting lipid hydroperoxide detoxification. With respect to inflammation-related DEGs, various proinflammatory cytokines were classified into cluster 20 in *F. nucleatum* group (such as *IL-1β*, *IL-6*, and *IL-8*), which showed a gradual increasing trend. The expression levels of these genes gradually increased during infection. Some of the genes were validated by qRT-PCR, and the expression levels were consistent with the RNA-Seq results (Fig. S7).

### Inflammatory genes are expressed sequentially in response to *F. nucleatum* infection

**Fig. 5:**
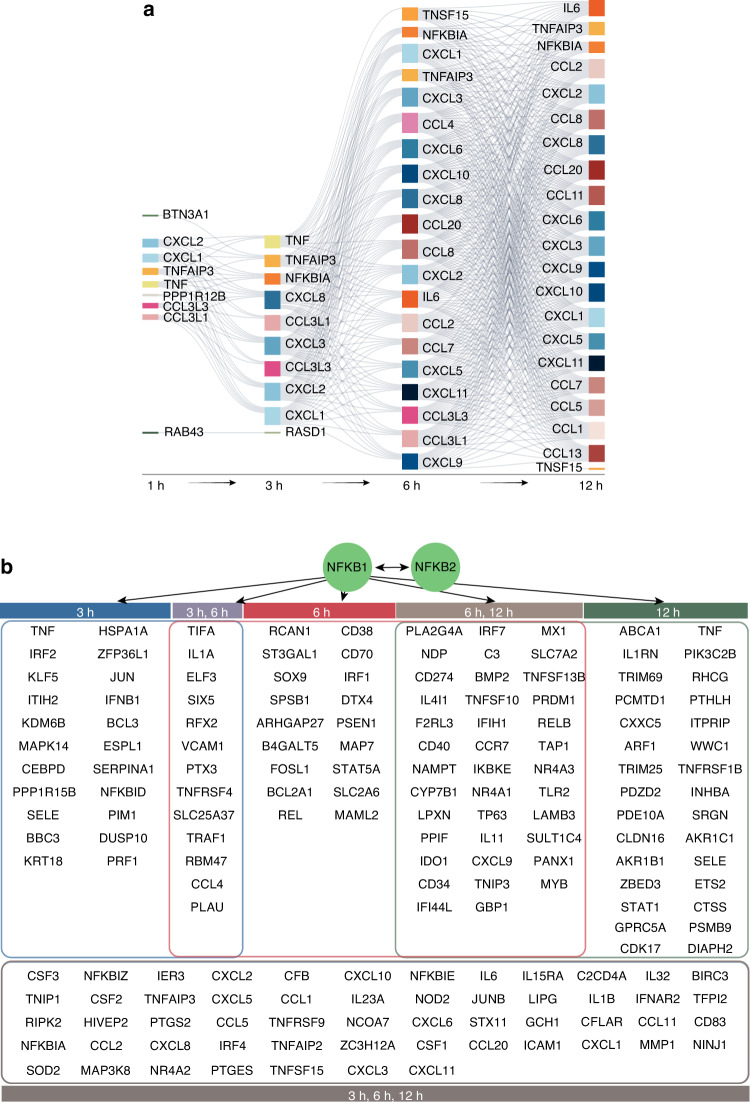
Gene regulation process in response to *F. nucleatum* infection in PDLSCs. **a** Coexpression network of DEGs showed the intracellular cascade induced by *F. nucleatum-*infection. (The figure only shows the results of inflammation-related genes.) **b** Transcription factors NFKB1 and NFKB2 up-regulated multiple DEGs

To explore the intracellular cascade induced by *F. nucleatum-*infection, we further analyzed the coexpressed genes at two adjacent time points. As shown in Fig. 5a, 9 members of the CXC chemokine subfamily, 11 members of the CC chemokine subfamily, and some proinflammatory cytokines formed a coexpression network. Interestingly, all of the chemokines in this network are inflammatory chemokines and are mainly involved in the recruitment of leukocytes to inflamed tissues.^28^ In addition, CCL11 and CCL20 have homeostatic functions and act as dual-function chemokines. The inflammatory chemokines CXCL1, CXCL2, CCL3L1, and CCL3L3 and inflammatory cytokine TNF were first released in response to *F. nucleatum* stimulation. With increasing duration of infection, the types and expression levels of chemokines increased gradually, which suggesting the potential of PDLSCs to recruit immune cells.

Additionally, several transcription factors were differentially expressed at 3 h and regulated a series of target genes among DEGs at 3 h, 6 h, and 12 h. Notably, NFKB1 and NFKB2, two central activators of genes involved in inflammation and immune function, were significantly upregulated at 3 h after *F. nucleatum*-infection and showed sustained activation at 6 h and 12 h. The increases in NFKB1 and NFKB2 sequentially regulated downstream target genes of the NF-κB signaling pathway, such as *MAP3K8*, *NFKBIA*, and *REL* (Fig. 5b).

**Fig. 6:**
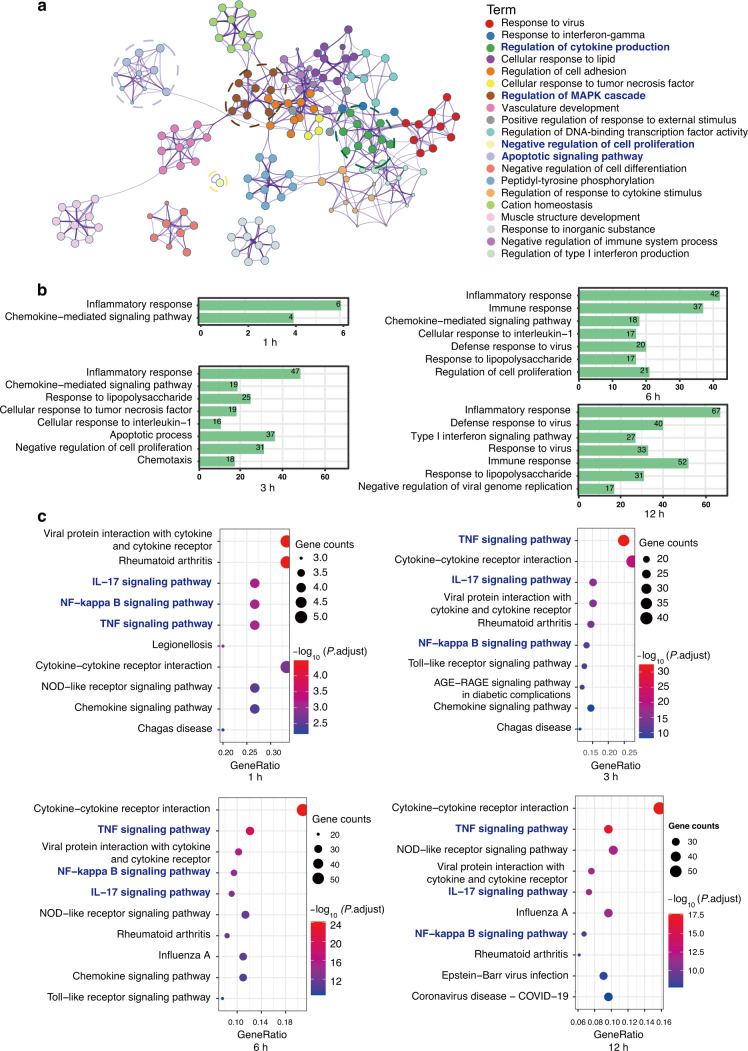
GO and KEGG pathway enrichment analysis of differentially expressed genes. **a** Network of enriched terms colored by cluster identity. **b** GO biological process analysis of DEGs. **c** KEGG pathway analysis of DEGs of 1 h, 3 h, 6 h, and 12 h

To improve our understanding of the biological functions of DEGs, we further performed Metascape analysis and displayed the top 20 enriched clusters in Fig. 6a. The regulation of cytokine production, the MAPK cascade, the apoptotic signaling pathway and the negative regulation of cell proliferation were highlighted in the network. Consistent with these results, GO analysis of the DEGs indicated that they were involved in the inflammatory response process (GO:0006954) and chemokine-mediated signaling pathway (GO:0070098) during *F. nucleatum* infection. Immune-related processes were changed in the initial phase of infection. With increasing duration of infection, genes were enriched in the apoptotic process (GO:0006915) and the negative regulation of cell proliferation (GO:0008285) (Fig. 6b and Fig. S8).

The top 10 abundant KEGG pathways are displayed in Fig. 6c, which shows that *F. nucleatum* stimulation mainly resulted in changes in immune-related pathways. DEGs at 1 h were mainly enriched in the cytokine receptor pathway, while at the three other time points, the IL-17, TNF and NF-κB signaling pathways were prominent. Pathview analysis showed the activated genes in the TNF, IL-17, and NF-κB signaling pathways. Most of the significantly altered genes were associated with survival and inflammation (Fig. S9). These findings suggested that the reactions of PDLSCs to *F. nucleatum* infection involved the recognition of bacterial surface epitopes by host receptors at 1 h, followed by activation of the host defense system within 12 h after infection. These results collectively indicated that *F. nucleatum* could induce an inflammatory response in PDLSCs associated with activation of the NF-κB signaling pathway and the production of inflammatory chemokines.

### Screening miRNAs and transcription factors and constructing the gene regulatory network of PDLSCs

**Fig. 7:**
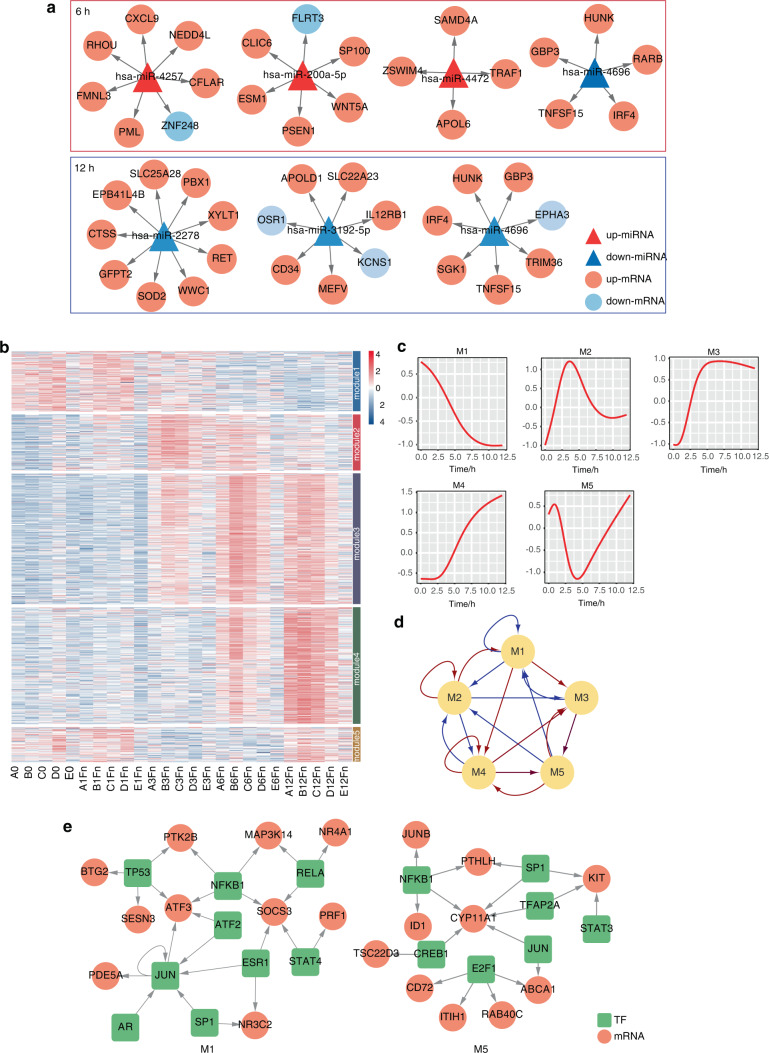
Construction of miRNA network and transcription factors network. **a** miRNA-mRNA regulatory network. **b** K-means method clustered the DEGs into 5 modules. **c** Gene expression trends in 5 modules. **d** The functional linkages between the modules. Red arrows represent positive regulation, and blue arrows represent negative regulation. **e** Regulatory networks of module 1 and module 5

Next, we sought to identify the miRNAs and their target mRNAs that were specifically expressed during *F. nucleatum* infection as described in the Materials and Methods. The differentially expressed miRNAs at each time point are listed in Table S4. After matching the differentially expressed miRNAs with their predicted target genes, we constructed networks containing 3 upregulated miRNAs and 1 downregulated miRNA associated with a total of 22 target genes at 6 h, and 3 downregulated miRNAs associated with 23 target genes at 12 h (Fig. 7a). Notably, target genes of miR-4257 were significantly enriched in cysteine-type endopeptidase activity involved in apoptotic process; target genes of miR-4696 were significantly enriched in iron ion homeostasis and positive regulation of MAPK cascade (Fig. S10).

Next, we clustered the DEGs by the similarity of expression patterns to investigate gene regulation during *F. nucleatum* infection. As shown in Fig. 7b, all DEGs were grouped into 5 clusters, which were named Module 1- Module 5 (M1-M5): (1) genes in Module 1 were continuously downregulated during *F. nucleatum* infection; (2) genes in Module 2 were upregulated in the early stages of *F. nucleatum* infection and downregulated at the following time points; (3) genes in Module 3 was gradually up-regulated within 6 h after infection; (4) genes in Module 4 were upregulated after 1 h; and (5) and genes in Module 5 were downregulated between 1 h and 3 h and recovered after 3 h (Fig. 7c). To reveal the overall regulatory relationships of the *F. nucleatum*-infected PDLSCs, we constructed a regulatory network between modules using high-dimensional ordinary differential equations,^29^ as shown in Fig. 7d.

At the functional level, GO analysis confirmed that genes in M1 were involved in signal transduction, genes in M5 were enriched in activating G-protein coupled receptor signaling pathway, and genes in M4 were involved in the defense response to virus and the innate immune response. To decipher the inter-module regulatory relationships, we integrated the regulatory linkages between the TFs and their target genes, and constructed regulatory networks (Fig. 7e and Fig. S11). Consistent with previous results, NFKB1 was predicted in all of the modules. Taken together, these results indicate the pivotal role of NFKB1 and revealed the gene regulation process at different time points of *F. nucleatum* infection.

### Identification of the therapeutic targets to attenuate the negative effects of *F. nucleatum* infection

**Fig. 8:**
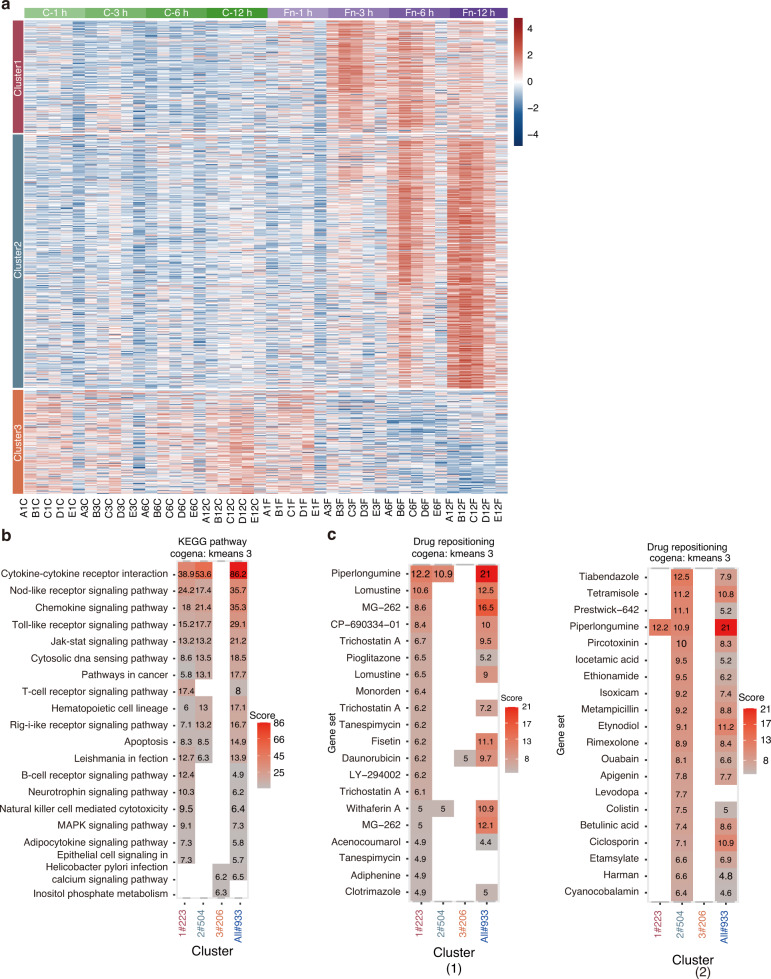
Drug repositioning based on transcriptome revealed candidate drugs. **a** K-means method clustered the DEGs of 1 h, 3 h, 6 h and 12 h into 3 clusters. **b** KEGG pathway analysis for coexpressed genes generated by cogena. **c** Computational drug repositioning for the coexpressed genes. Drug candidates target DEGs in (1) cluster 1 and (2) cluster 2

We used cogena^30^ to perform coexpression analysis and divided the DEGs into three clusters (Fig. 8a). KEGG pathway enrichment analysis of the coexpressed genes showed that genes in clusters 1 and 2 were highly enriched in immune-related pathways, while genes in cluster 3 were enriched in calcium signaling pathway and inositol phosphate metabolism (Fig. 8b). Considering the major pathological changes in *F. nucleatum*-infected PDLSCs, we further performed drug repositioning analysis of clusters 1 and 2 to identify potential drug candidates. The list of drug candidates targeting the coexpressed genes in clusters 1 and 2 is shown in Fig. 8c. Pathway enrichment analysis of the target genes of each candidate drug was performed to narrow the field of candidates (Fig. S12). Based on the enrichment results, we ultimately selected six drugs and assessed the therapeutic value of these six candidates.

Cytotoxicity assays helped us to select the drug concentrations that cells could tolerate for the follow-up experiments (Fig. S13a). After 12 h of *F. nucleatum* infection, PDLSCs had significantly elevated mRNA expression of *IL1β*, *IL6* and *IL8* (*P* < 0.05), and all six candidates significantly reduced the expression level of these inflammatory genes (*P* < 0.05) (Fig. S13b). These results validated the efficacy of our predicted agents.

**Fig. 9:**
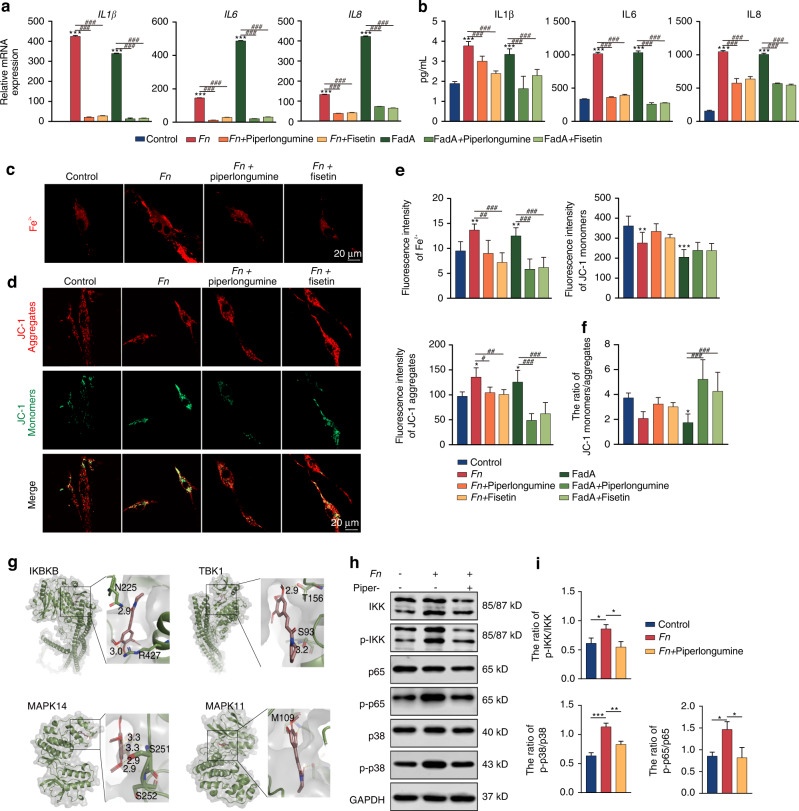
Experimental validation of the candidate drugs. **a** QRT-PCR and **b** ELISA showed piperlongumine and fisetin suppressed the expression of inflammatory cytokines. **c** Piperlongumine and fisetin reversed the increased intracellular Fe^2+^ induced by *F. nucleatum* stimulation. Scale bar: 20 μm. **d** Effect of piperlongumine and fisetin on the mitochondrial membrane potential. Scale bar: 20 μm. **e** Quantitative assessment of FerroOrange and JC-1 fluorescence. **f** The ratio of JC-1 monomers/aggregates. **g** Molecular docking between piperlongumine and main targets. **h** Effects of piperlongumine on the phosphorylation of IKK, p38, and NF-κB p65 detected by Western blot analysis. **i** Quantitative analysis of altered protein expression of these proteins used ImageJ. Data were expressed as mean ± SD. (*n* = 3) (*P < 0.05; **P < 0.01; ***P < 0.001, compared with the control group; ^#^*P* < 0.05; ^##^*P* < 0.01; ^###^*P* < 0.001)

Among the drugs investigated, piperlongumine and fisetin exhibited the best attenuating effects, which prompted us to further assess their effects on FadA-induced inflammation. Similarly, piperlongumine and fisetin significantly decreased the FadA-induced proinflammatory cytokine production (*P* < 0.001) (Fig. 9a, b). Considering the ferroptotic effects of *F. nucleatum* on PDLSCs, we further examined the effects of piperlongumine and fisetin on ferroptosis. As shown in Fig. 9c–f, piperlongumine and fisetin reversed this trend, reduced the level of intracellular Fe^2+^, and ameliorated the impairment in mitochondrial function. As the IL-17 signaling pathway and NF-κB signaling pathway were enriched by KEGG analysis, we used molecular docking to simulate the binding of piperlongumine or fisetin with key protein targets of the IL-17 and NF-κB signaling pathways. The predicted hub targets of piperlongumine are displayed in the 3D results in Fig. 9g. To validate these prediction results, we next evaluated the effects of piperlongumine on the *F. nucleatum*-induced expression of downstream markers. Western blot analysis showed that *F. nucleatum* infection increased the phosphorylation of IKK, p65 and p38, and piperlongumine treatment significantly inhibited the F. nucleatum-induced activation of IKK, p65 and p38 (Fig. 9h–i).

## Discussion

Recent studies indicate that PDLSCs play a crucial role in the maintenance of periodontal homeostasis.^31^The normal periodontal milieu is in a dynamic equilibrium of cell proliferation and apoptosis,^32^ and the invasion of periodontal pathogens could impair the self-renewal function of PDLSCs.^33^ In our study, we demonstrated that *F. nucleatum* inhibited cell proliferation and promoted apoptosis in PDLSCs and first showed that *F. nucleatum* could induce ferroptosis by intervening in iron metabolism in PDLSCs. As a bacterial virulence strategy, programmed cell death in response to bacterial infection is a complex process, involved in apoptosis, pyroptosis, necroptosis and ferroptosis.^34^ Further studies are needed to illuminate the intersections between apoptosis and ferroptosis, or with other programmed cell death pathways.

FadA was reported to mediate the pathogenic effect of *F. nucleatum* on colorectal cancer cells.^16^ In this study, we showed that FadA acted as a virulence factor and increased the expression levels of inflammatory cytokines in PDLSCs. We first identified PEBP1 as a FadA-interacting protein and showed that binding with FadA could deactivate PEBP1 to activate the IKK-NF-κB and Raf1-MAPK signaling pathways. It has been reported that PEBP1 is involved in inflammation-related diseases,^35^ including autoimmune diseases^36^ and antiviral innate immune responses.^37^ Our findings further confirmed the pivotal effect of PEBP1 on inflammation, and revealed a previously unrecognized molecular mechanism of *F. nucleatum* pathogenicity.

Cytokine secretion is the first wave of the host immune response to periodontal pathogen challenge.^38^ Our study showed that *F. nucleatum* infection stimulated the secretion of IL-1β, IL-6, and IL-8 in the very early stage. The results of RNA-seq also proved that ample cytokines and chemokines were released in the initial stage of *F. nucleatum* infection. These findings proved that PDLSCs have immunoregulatory capacity and that *F. nucleatum* could aggravate periodontal inflammation by impairing the immunosuppressive function of PDLSCs. However, the prolongation of *F. nucleatum* infection did not lead to the continuous secretion of these inflammatory cytokines. This is probably because of the limitations of the in vitro cell model as it cannot perfectly replicate the conditions found within living organisms. In addition, *F. nucleatum* has limited survival time in the aerobic environment,^39^ which may also limit its pathogenicity.

Drug repositioning is a tool for exploring new uses for approved or investigational drugs. The coexpression-based drug repositioning prediction and experimental validation findings suggest that piperlongumine and fisetin could be candidate drugs to treat *F. nucleatum*-infected PDLSCs. Our results are important in identifying drugs to treat periodontal pathogen infection, but the evaluation of drug effects requires more profound and systematic research.

In summary, our study provides more specific evidence of the host’s early immune response to *F. nucleatum* infection and provides novel insight into the pathogenic mechanism of periodontitis.

## Materials and methods

### Bacteria and cell culture

This study was approved by the Ethics Committee of Stomatology Hospital of Shandong University (Protocol Number: 20170303) and all volunteers signed the informed consent before providing the oral tissue samples. *Fusobacterium nucleatum* ATCC 25586 was provided by the Shandong Key Laboratory of Oral Tissue Regeneration (Jinan, China). Human PDLSCs obtained from healthy premolars and third molars were freshly extracted as described in the previous study.^40^

### Cell phenotype analysis and multilineage differentiation assays

To identify the cell phenotype of PDLSCs, BD Stemflow^TM^hMSC Analysis Kit (BD Biosciences, California, USA) was implemented according to the manufacturer’s instructions. For multilineage differentiation assays, PDLSCs were cultivated in 6-well plates at 2 × 10^5^ cells per well. At 80%–90% density, the corresponding differentiation medium was replaced to assess osteogenesis and adipogenesis. After 21 days, the cells were stained by Alizarin Red (Sigma‐Aldrich, Missouri, USA) to observe the mineralization. After 28 days, the adipogenesis was detected by Oil Red O (Sigma) staining.

### Cell viability assays and cell apoptosis analysis

The number of cells was counted using Countstar (Shanghai, China). The proliferation rate was detected using an EdU Apollo DNA in vitro kit (RiboBio, Guangzhou, China) and observed by a fluorescent microscope. Cell viability was estimated by Cell Counting Kit-8 (Boster, Wuhan, China). In accordance with the manufacturer’s instructions, Annexin V-FITC/PI double staining kit (Dojindo, Kumamoto, Japan) was used to detect apoptosis.

### Enzyme-linked immunosorbent assay (ELISA)

The proinflammatory cytokine concentrations were evaluated using the specific ELISA kits (Biolegend, California, USA). The optical density values were measured at 450 nm and 570 nm by a microplate reader.

### Real-time quantitative PCR Analysis

Total RNA was isolated with TRIzol reagent (CWBIO, Beijing, China), and cDNA was reverse transcribed using a HiFiScript cDNA Synthesis kit (CWBIO). Real-time quantitative PCR (qRT-PCR) was performed using UltraSYBR Mixture (CWBIO). The relative mRNA expression levels were analyzed by the 2^^(-ΔΔct)^ method and normalized by the GAPDH level. The sequences of the primers used in the experiment are listed in Table S1.

### Western Blot analysis

Cells were lysed in a RIPA lysis containing PMSF (Solarbio, Beijing, China). Protein concentration was measured with a BCA Protein Assay Kit (Cwbio). Equivalent amounts of proteins were loaded onto SDS-PAGE gels and transferred to PVDF membranes (Millipore, Massachusetts, USA). After blocked with 5% milk in TBST and incubated with primary antibodies listed in Table S2 overnight, the membranes were incubated with HRP-conjugated secondary antibodies (Proteintech, Indiana, USA). The immunoreactive bands were visualized by an Immobilon Western HRP Substrate (Millipore) and determined using ImageJ gel analysis software.

### Detection of intracellular Fe^2+^ amount and Mitochondrial Membrane Potential (MitoMP) Assessment

Intracellular Fe^2+^ levels were examined using FerroOrange (Dojindo) according to the manufacturer’s instructions. Mitochondrial membrane potential was detected by a MitoMP assay Kit with JC-1 (Solarbio). The fluorescent intensity was measured using the EnVision multimode microplate reader (PerkinElmer, Massachusetts, USA). The fluorescence images were obtained by Dragonfly 200 high speed confocal microscope (Andor Technology, Belfast, UK).

### Recombinant protein production and purification

FadA and PEBP1 were purified as previously described.^17,41^ The entire *fadA* gene of *F. nucleatum* ATCC 25586 and the entire *pebp1* gene of *Homo sapiens* were synthesized by Sangon Biotech (Shanghai, China). After verification, the prokaryotic expression vector was transformed into *E. coli* BL21(DE3). *E. coli* was grown in LB medium to an OD_600_ of 0.6. Then the cultures were induced by 0.5 mmol·L^-1^ isopropyl β-d-1-thiogalactopyranoside (IPTG) (Aladdin, Shanghai, China) for 2.5 h. An His-tag Protein Purification Kit (Byotime, Shanghai, China) was used for FadA purification, and Amicon® Ultra-15 Centrifugal Filters (Millipore) were used for desalting, diafiltration and concentrated. The concentration of FadA for further cellular experiments was chosen based on the concentrations reported in the literature.^16^

### Pull-down assay

The specific method of His pull-down refers to Pierce pull-down polyhis protein: protein interaction kit (Thermo Fisher Scientific, MA, USA). The pull-down proteins were digested into proteolytic peptides and identified using Liquid chromatography and mass spectrometry (Thermo Fisher Scientific).

### Co-immunoprecipitation (Co-IP) assay

To check whether FadA/PEBP1 interaction occurs in vivo, PDLSCs were preincubated with FadA. Total protein from PDLSCs was extracted using NP-40 solution (Boster) containing 1 mmol·L^-1^ PMSF. A total of 1 000 μg of cell lysate was incubated with 5 μg anti-His antibody (Proteintech) or IgG (Santa Cruz Biotechnology, Texas, USA) at 4 °C overnight. The protein complex was captured overnight by Protein A/G agarose (Santa Cruz Biotechnology) at 4 °C. The beads were collected by centrifugation at 12 000 × *g*, followed by 3 washes and Western blot analysis.

### Surface plasmon resonance (SPR) analysis

We performed SPR analysis using a Biacore T200 (GE Healthcare, MA, USA). Approximately 200 RU of His-tagged recombinant FadA was immobilized on a sensor chip CM5 using amine coupling chemistry. Unreacted moieties were blocked with ethanolamine. Recombinantly purified PEBP1 was passed over the sensor chip in different concentrations from 0 μmol·L^-1^ to 640 μmol·L^-1^, with the 40 μmol·L^-1^ concentration as internal control. All binding curves were normalized to a baseline of 0 and the reference flow cell value was subtracted.

### RNA-sequencing analysis

A total of 45 samples from 5 individuals co-cultured without or with *F. nucleatum* at an MOI of 100) for 0, 1, 3, 6, and 12 h were analyzed by RNA-Sequencing (RNA-seq) at LC-BIO (Hangzhou, China). The raw sequence data reported in this paper have been deposited in the Genome Sequence Archive (Genomics, Proteomics & Bioinformatics 2021)^42^ in National Genomics Data Center (Nucleic Acids Res 2022),^43^ China National Center for Bioinformation/Beijing Institute of Genomics, Chinese Academy of Sciences (GSA-human: HRA002672) that are publicly accessible at https://ngdc.cncb.ac.cn/gsa-human. R package DESeq2 (version)^44^ was used for screening differential expression genes by setting statistical significance value (*P*-value) <0.01 and absolute value of log_2_ (fold change) >1. We used R package Mfuzz (version 2.50.0)^45^ to classify the gene expression clusters. DAVID^46^ and R package clusterProfiler (version 3.18.1)^47^ were used for GO and KEGG enrichment analysis. R Pathview (version 1.30.1)^48^ was used to visualize significant KEGG signaling pathways.

### Construction of miRNA-mRNA network and regulatory network

The has.gff3 annotation files were downloaded from the miRbase database, and the microRNA expression was obtained using FeatureCounts. R package DESeq2 (version) was used for screening differential expression miRNAs by setting statistical significance value (*P*-value) < 0.05. The potential target genes of miRNAs were predicted by miRWalk2.0. The miRWalk, miRanda, miRMap, and Targetscan database were added to help predicting supposed target genes.

The module regulatory relationships were calculated using the reported method.^29^ We used TRRUST^49^ to explore the TF targets and used RegNetwork^50^ to construct a TF-miRNA-gene regulatory network.

### Computational drug repositioning analysis

The drug repositioning for the coexpressed genes were performed using the cogena package (version 1.24.0).^30^ The SwissTargetPrediction database was used to predict potential effector targets.^51^ The SMILES format of candidate drugs obtained from ZINC15 was inputted into this database to obtain the potential effector targets of the candidate drugs.

We performed molecular docking using the program AutoDock Vina (version 1.1.2).^52^ The 3D structure of the candidate drugs was obtained from the ZINC15 and the structures of target proteins were obtained from PDB database or uniport database. AutoDockTools (version 1.5.6) was used to process the ingredients and protein structures. PyMOL (version 4.6.0) was used to visualize the combinations.

### Statistical analysis

All experiments were repeated independently at least three times with cells from three volunteers, and the data were plotted as mean ± standard deviation (SD). Data were analyzed using GraphPad Prism (version 8.0). Differences among multiple groups were assessed using one-way or two-way ANOVA followed by Tukey’s honestly significant difference comparison test. Differences were considered statistically significant at *P* < 0.05.

## Supplementary information

Supplementary figures

Supplementary tables
