# Supplementary material for: Study of the inflammatory activating process in the early stage of Fusobacterium nucleatum infected PDLSCs
Source: Int J Oral Sci. 2023 Feb 8;15:8. doi: 10.1038/s41368-022-00213-0 (PMC9908923; doi:10.1038/s41368-022-00213-0)
Supplement: Supplementary file 1 — Supplementary figures [file 41368_2022_213_MOESM1_ESM.docx]

**Supplementary Figures**


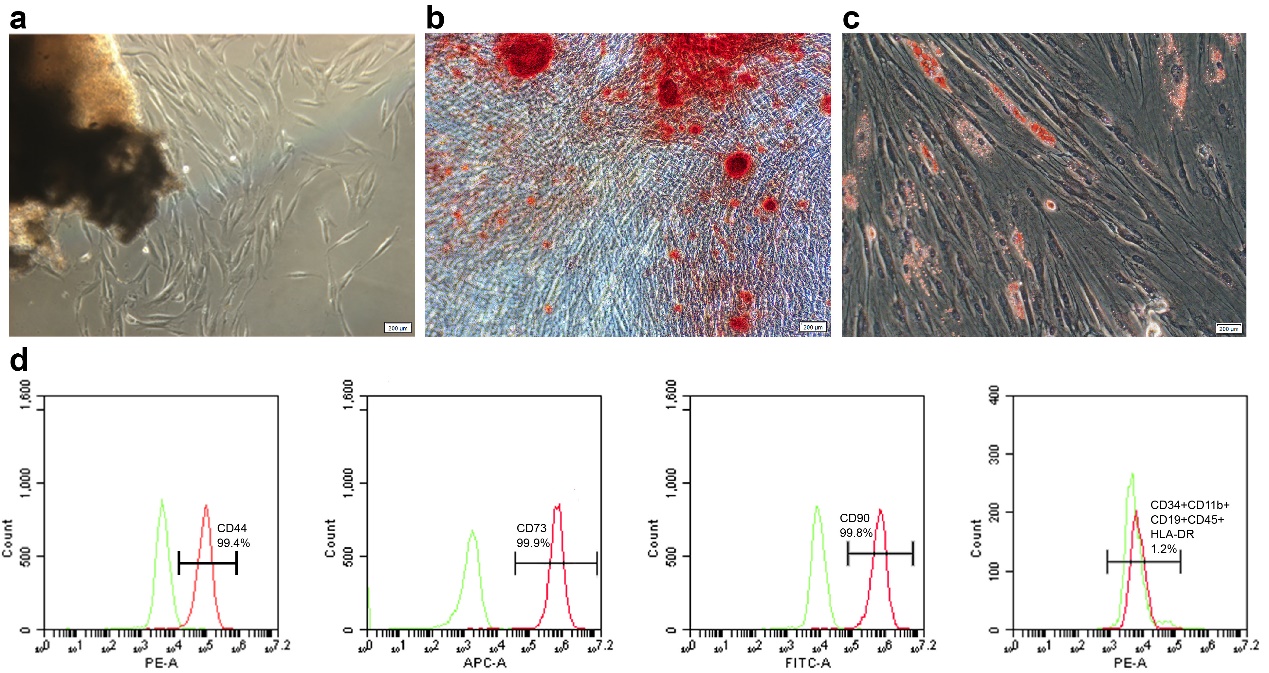


Fig. S1 Culture and identification of PDLSCs

(a) Morphology of primary PDLSCs. (b) Osteogenic differentiation of PDLSCs determined by Alizarin red S staining after 21 days of osteogenic induction. (c) Adipogenic differentiation of PDLSCs determined by Oil red O staining after 21 days of adipogenic induction. (d) Immunophenotype analysis of PDLSCs determined by Flow cytometry assay.


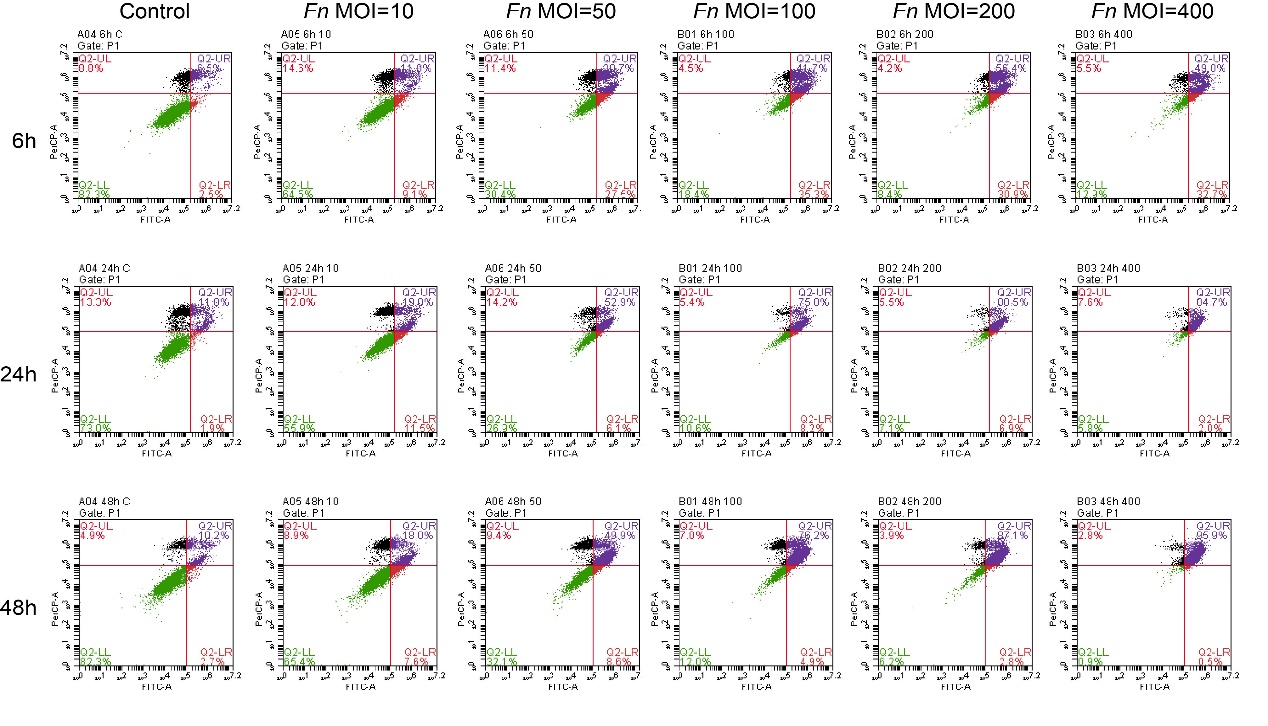


Fig. S2 Cell apoptosis analysis using flow cytometry of PDLSCs with or without *F. nucleatum* stimulation.


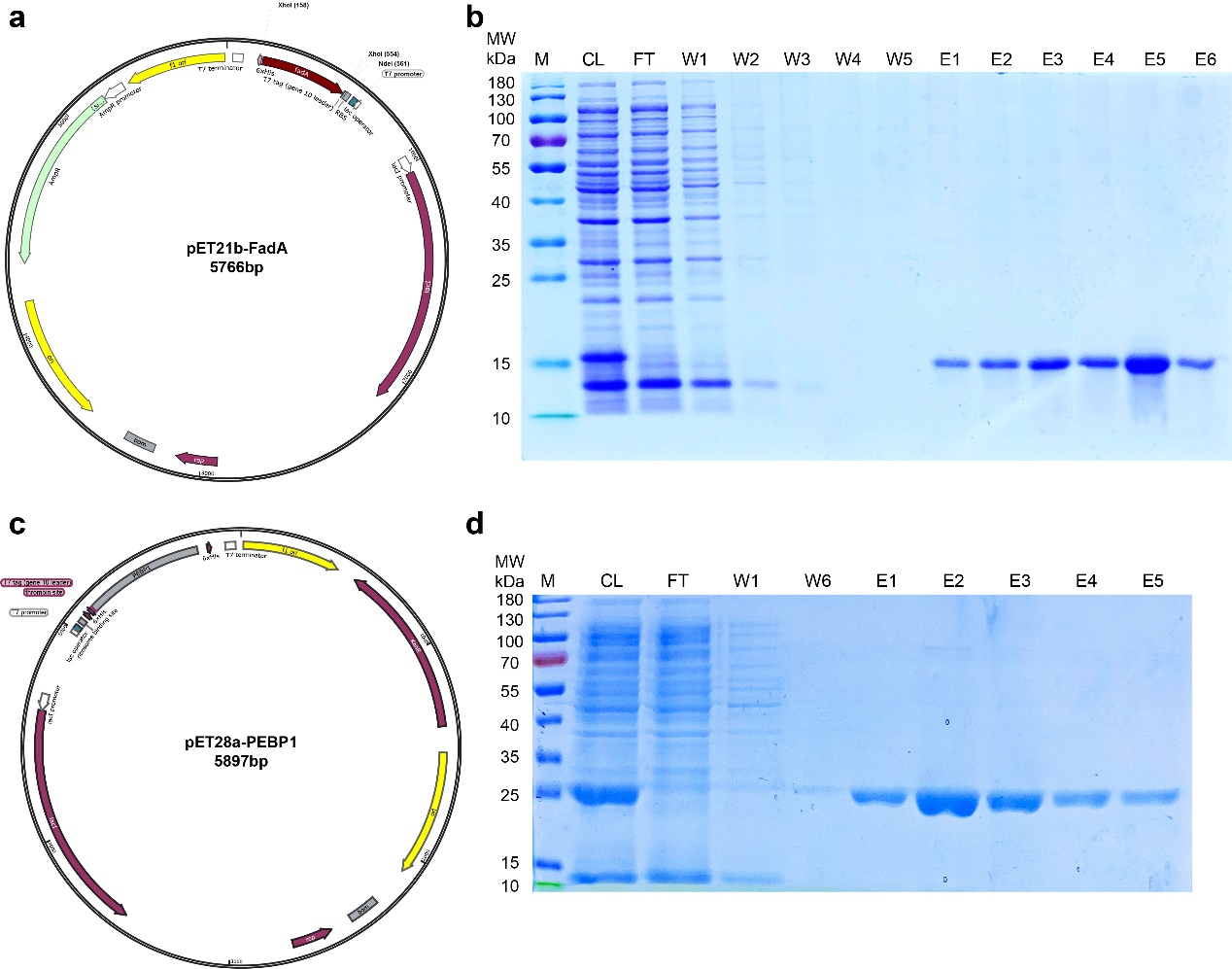


Fig. S3 Acquisition of heterogeneous expressed FadA protein

(a) Plasmid map of pET21b-FadA. (b) SDS-PAGE analysis of recombination protein expression in *E. coli* and purification. (c) Plasmid map of pET28a-PEBP1. (d) SDS-PAGE analysis of recombination protein expression in *E. coli* and purification. M: maker; CL: cell lysate; FT: flow through; W1-W5: wash 1-5; E1-6: elution 1-6.


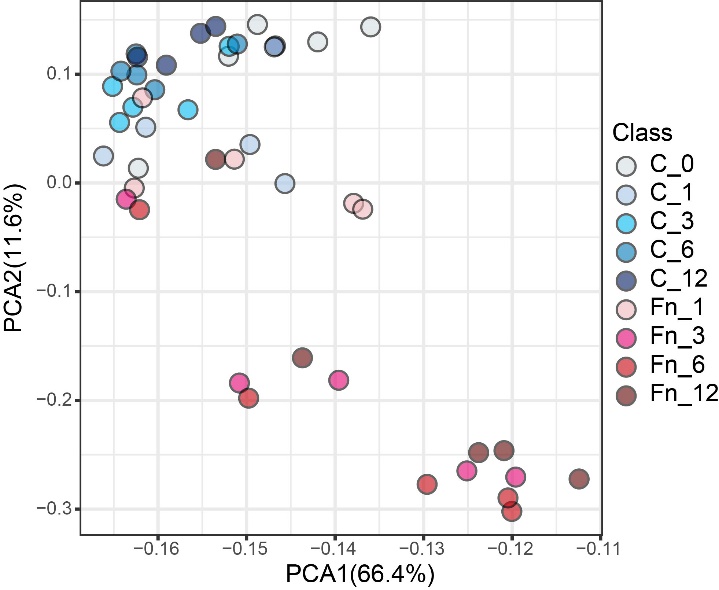


Fig. S4 PCA of 45 samples of RNA-seq analyses.


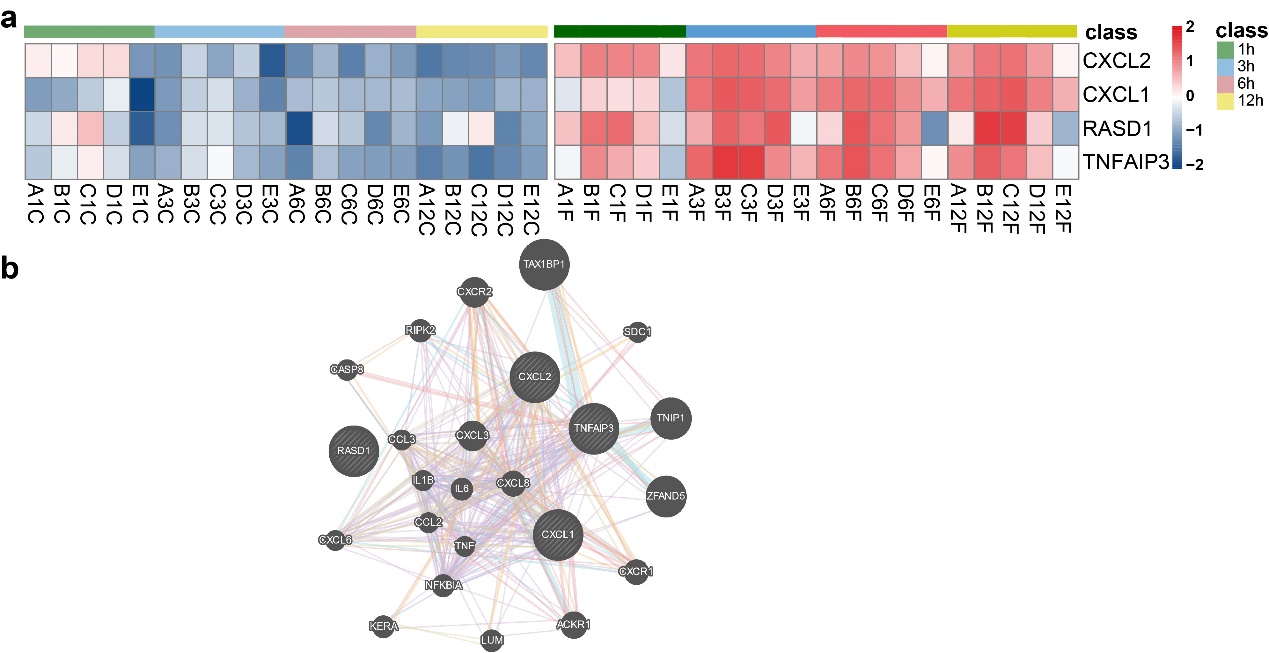


Fig. S5 (a) Heatmap of the average gene expression level of 4 overlapping DEGs. (b) The functional network constructed through GeneMANIA.


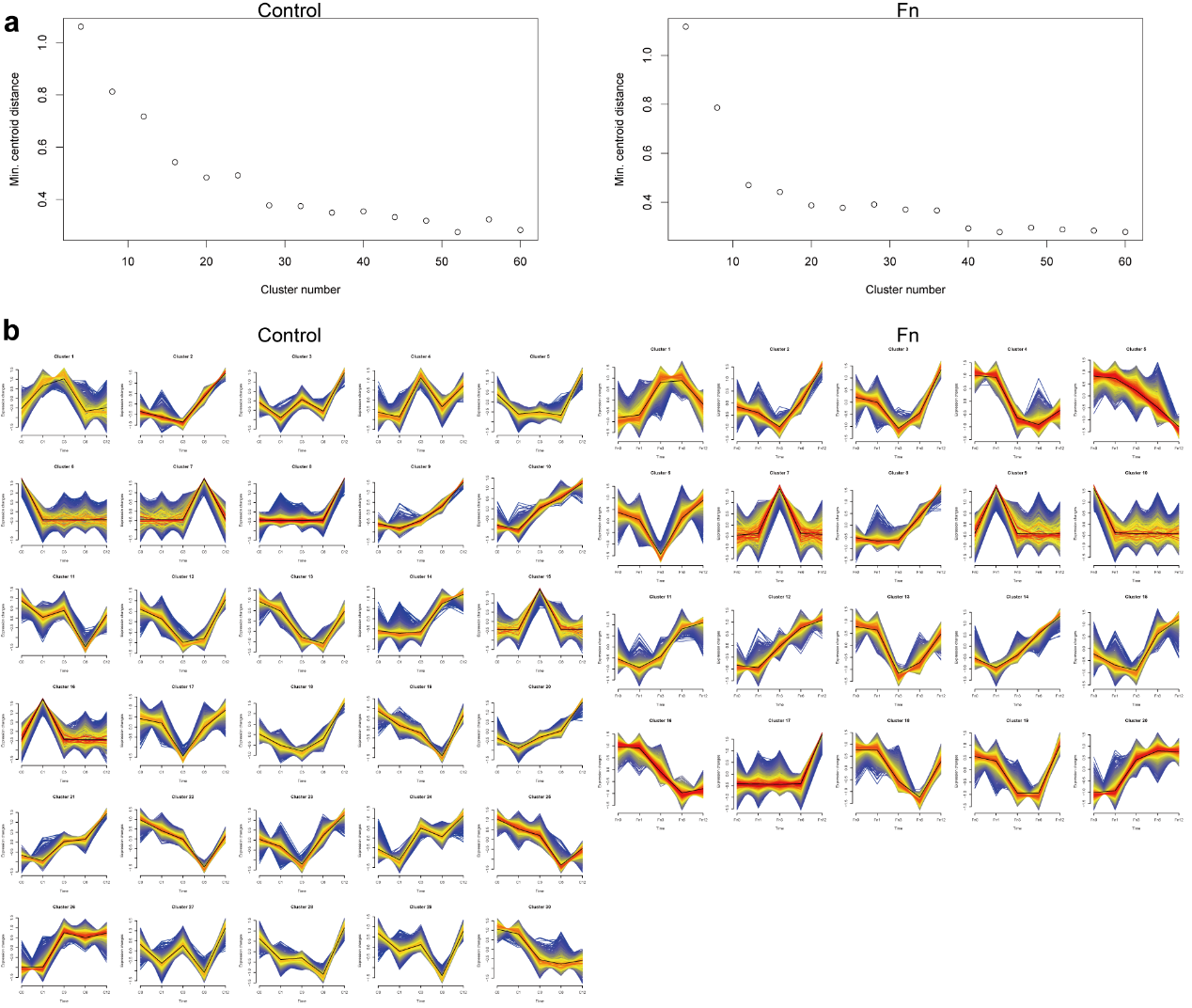


Fig. S6 Clustering of changes in gene expression in control and *F. nucleatum* group.


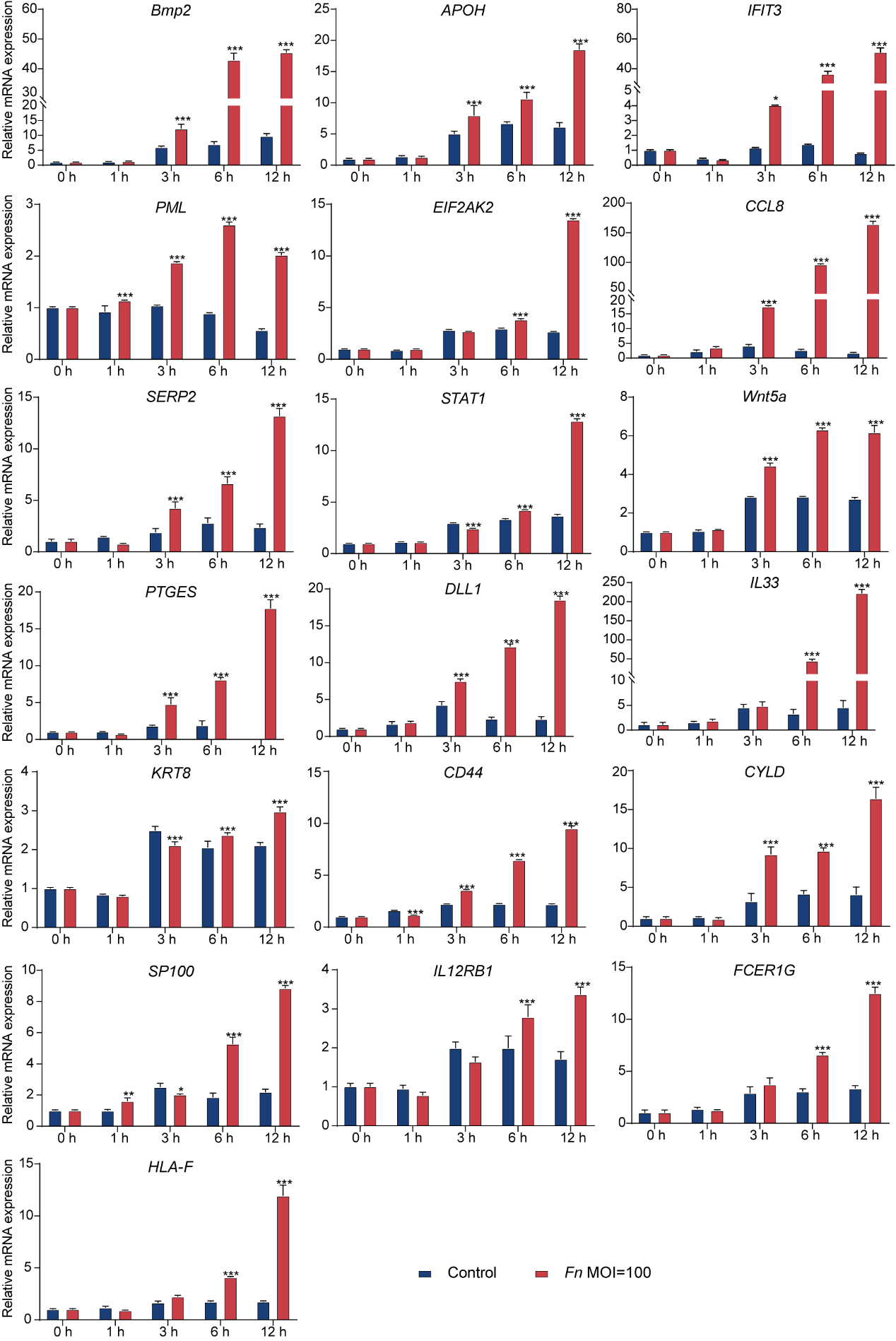


Fig. S7 Relative gene expression level detected by qRT-PCR. Data were expressed as mean ± SD. (*n*=3) (^*^*p* < 0.05; ^**^*p* < 0.01; ^***^*p* < 0.001, compared with the control group).
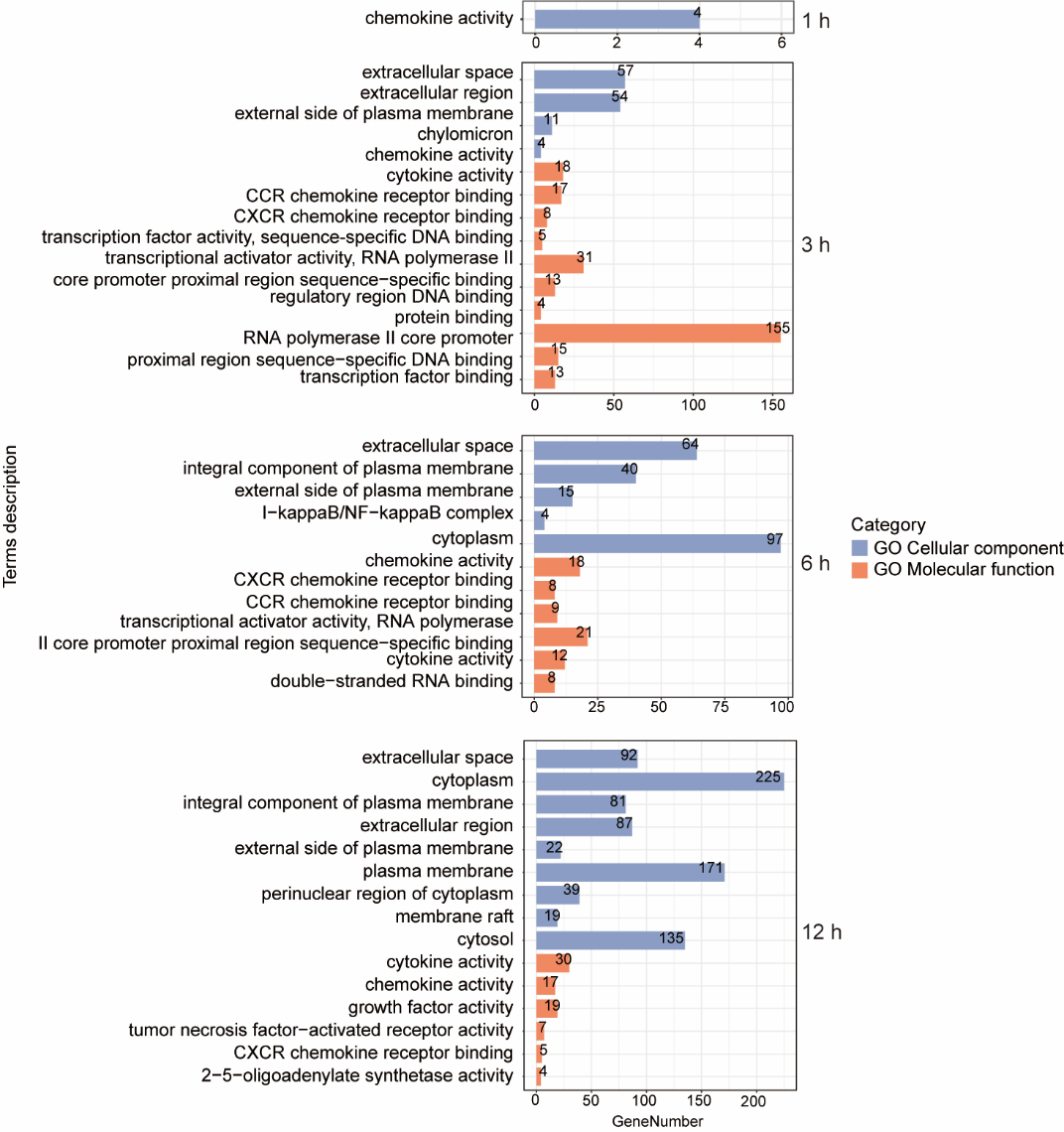


Fig. S8 GO cellular component analysis and GO molecular function analysis of DEGs.


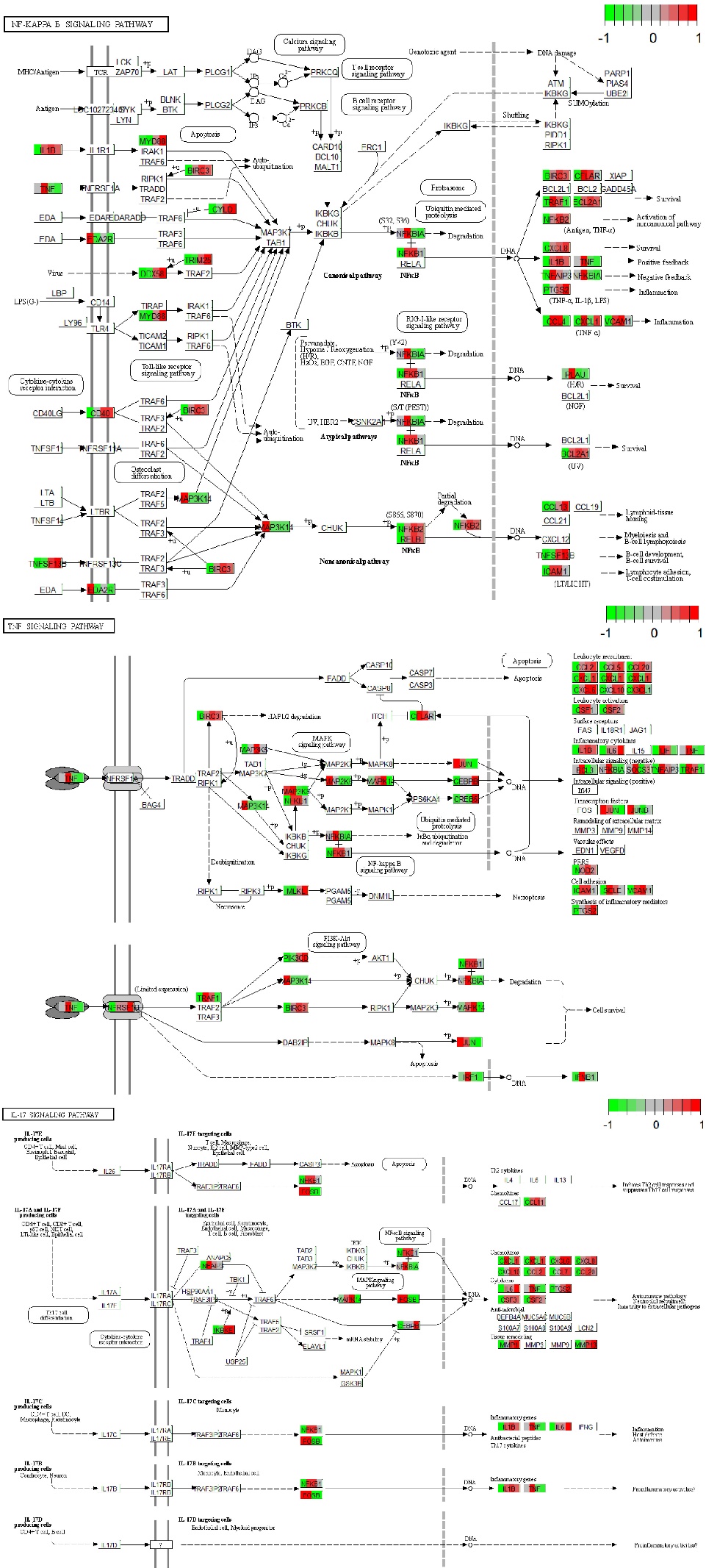


Fig. S9 The Pathview analysis of the (a) NF-κB, (b) TNF, and (c) IL-17 signaling pathways.


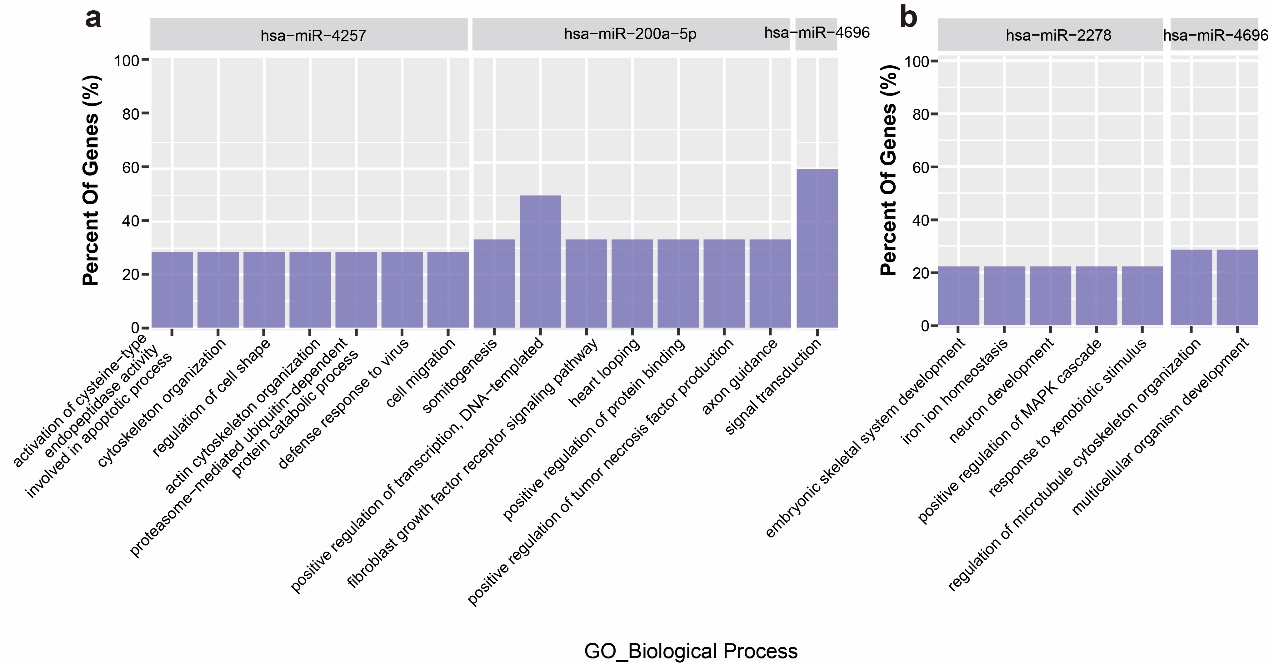


Fig. S10 Go enrichment of mRNA in the miRNA-mRNA regulatory network.


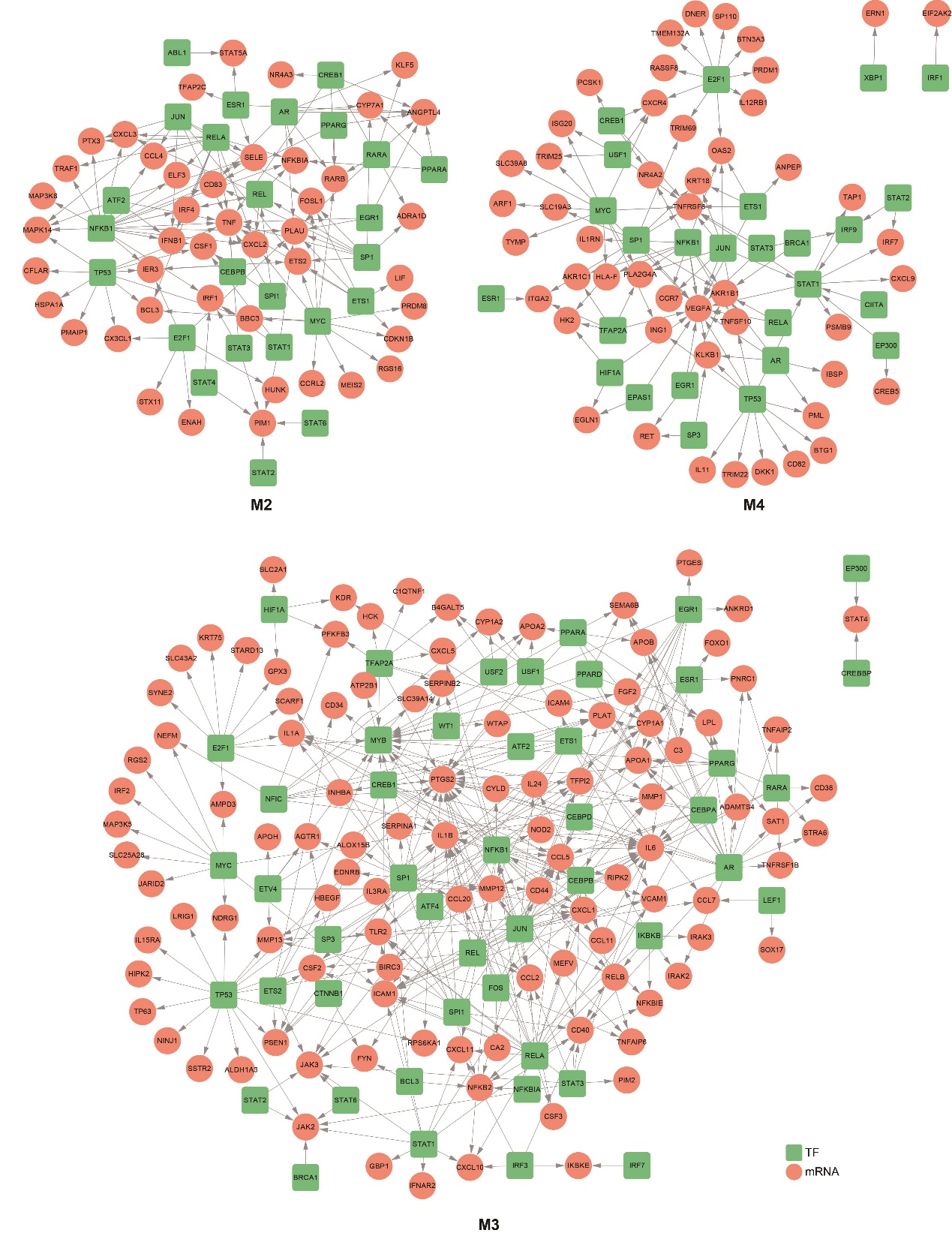


Fig. S11 Regulatory networks of module 2, module 3 and module 4.


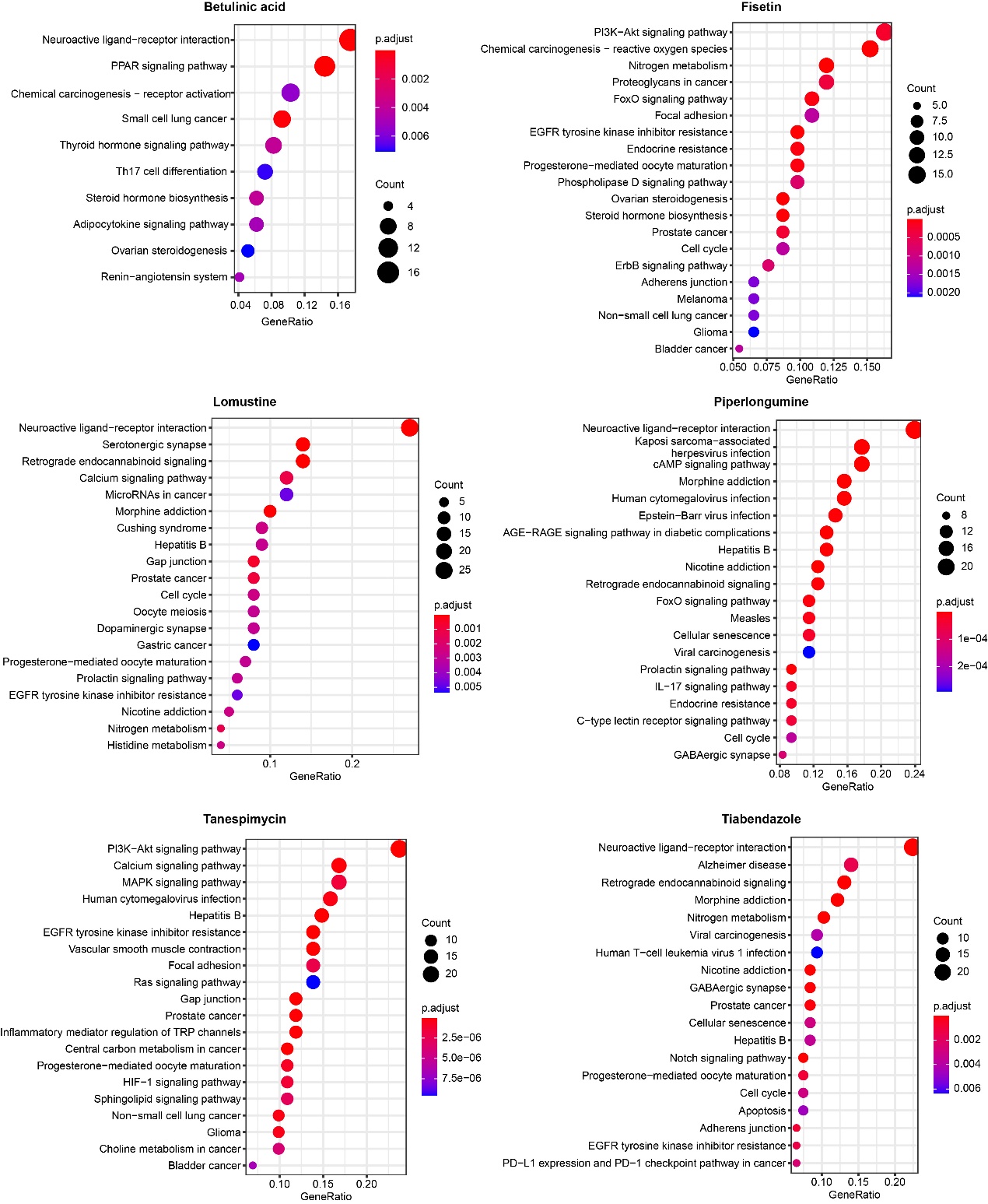


Fig. S12 GO enrichment analysis of drug candidates.


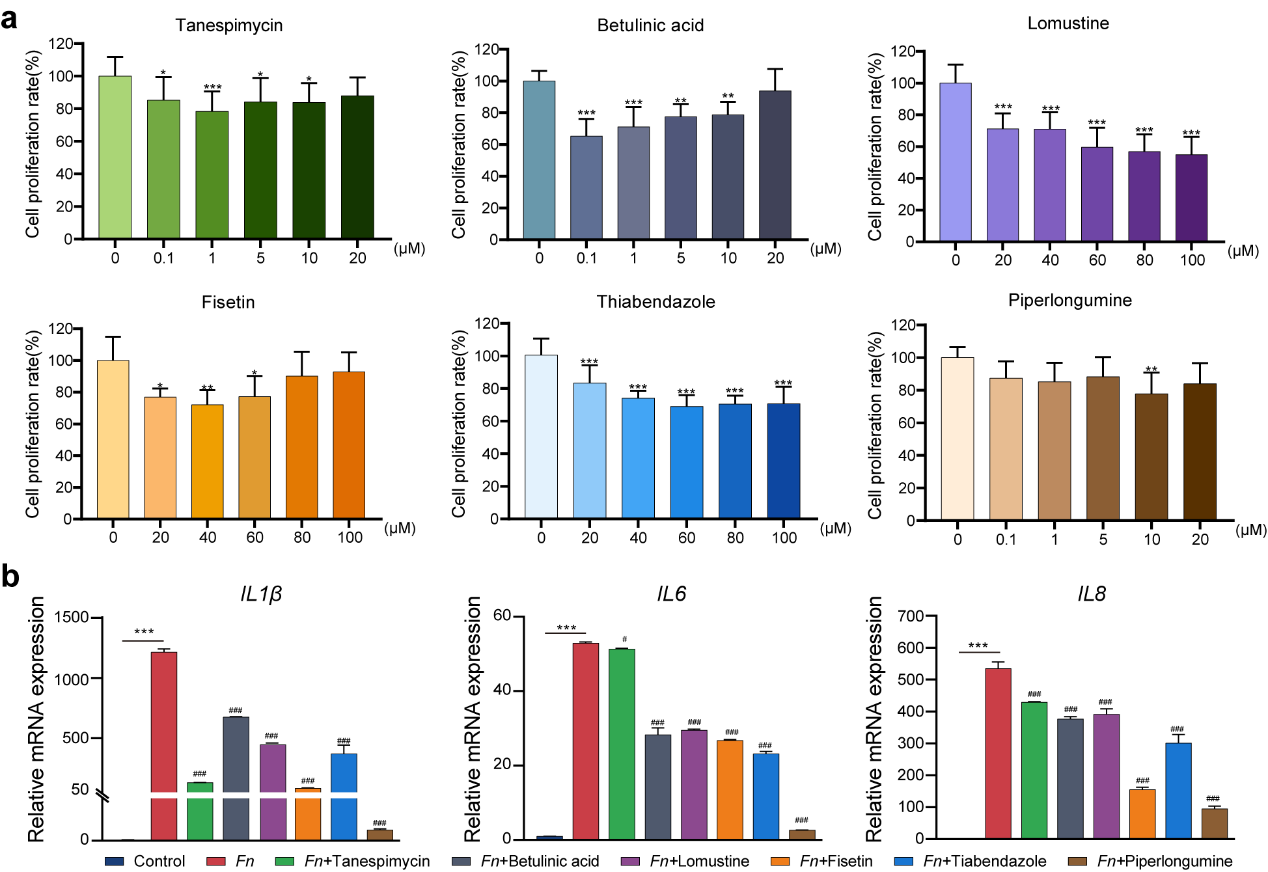


Fig. S13 (a) The proliferation rates of PDLSCs treated with drug candidates detected by CCK-8. (b) The anti-inflammation effect of candidate drugs detected by qRT-PCR. Data were expressed as mean ± SD. (*n*=3) (^*^*p* < 0.05; ^**^*p* < 0.01; ^***^*p* < 0.001, compared with the control group).
