# Supplementary material for: Study of the inflammatory activating process in the early stage of Fusobacterium nucleatum infected PDLSCs
Source: Int J Oral Sci. 2023 Feb 8;15:8. doi: 10.1038/s41368-022-00213-0 (PMC9908923; doi:10.1038/s41368-022-00213-0)
Supplement: Supplementary file 2 — Supplementary tables [file 41368_2022_213_MOESM2_ESM.docx]

**Supplementary Tables**

Table S1 Primers used in this experiment

| Names | Sequences | |
| --- | --- | --- |
|  | 5’-3’ Forward | 5’-3’Reverse |
| GAPDH | GCACCGTCAAGGCTGAGAAC | TGGTGAAGACGCCAGTGGA |
| Il-1β | CTTGAAGCTGATGGCCCTAAA | AGTGGTGGTCGGAGATTCGT |
| IL-6 | ATAACCACCCCTGACCCAAC | CCCATGCTACATTTGCCGAA |
| IL-8 | TCAGAGACAGCAGAGCACAC | GGCAAAACTGCACCTTCACA |
| KRT8 | GGAAGGGCTGACCGACGAG | CCAGGGAGCGGCTGTTGT |
| SFRP2 | ATCCTGGAGACCAAGAGCAAGAC | TGACCAGATAGGGCGCGTTGATG |
| CD44 | GCAAACACAACCTCTGGTCC | CCCACACCTTCTTCGACTGT |
| PML | CCCGCAAGACCAACAACATC | ACAGCGGCTTGGAACATCCT |
| IL33 | AAATGAATCAGGTGACGGTGTT | CCACAGAGTGTTCCTTGTTGTTG |
| CYLD | GAAGGTCGTGGTCAAGGT | GAATCTGTTCTCGGTGGT |
| SP100 | GCACACAGCCACGATTTG | CAGGTTAAATGTCTTCTC |
| WNT5A | CAACTGGCAGGACTTTCTCAA | CCTTCTCCAATGTACTGCATGTG |
| CCL8 | TGGAGAGCTACACAAGAATCACC | TGGTCCAGATGCTTCATGGAA |
| IL12RB1 | GCTGTACACTGTCACACTCTG | AACTTGGACACCTTGATGTCTC |
| FCER1G | GCCTGCATGCCATTAACACC | AACAGGGAGGAGGAACCACT |
| HLA-F | ATGCAGAGGAGTTCAGGACC | TCTGTGTCCTGGGTCTGTTC |
| STAT1 | CCAAAGGAAGCACCAGAGCC | AGAGCCCACTATCCGAGACACC |
| APOH | CCACTTTGGTAGTGCCAGTG | GGCCACAGTCCTGTGAGAG |
| EIF2AK2 | CAGAATTGACGGAAAGACTTACG | CTCTCAAGAGAATCATCACTGGT |
| DLL1 | CCGGTACTGTGACGAGTGTA | GTGGCTCCATTCTTGCAGG |
| IFIT3 | AACTACGCCTGGGTCTACTATCACTT | GCCCTTTCATTTCTTCCACAC |
| PTGES | CATGTGAGTCCCTGTGATGG | CTGCAGCAAAGACATCCAAA |
| BMP2 | GGCTGACCTGAGTGCCTGCGATA | GTCCTGAGCGAGTTCGAGTTGCG |

Table S2 Antibodies used in this experiment

| **Antibody** | **Company** | **Dilution** |
| --- | --- | --- |
| Raf1 | Abcam | 1:1000 |
| Phospho-Raf1 (S259) | Abcam | 1:1000 |
| IKKα/β | Beyotime | 1:1000 |
| Phospho-IKKα/β (S176/180) | Beyotime | 1:1000 |
| NF-κB p65 | Cell Signaling Technology | 1:1000 |
| Phospho-NF-κB p65 (S536) | Cell Signaling Technology | 1:1000 |
| JNK | Cell Signaling Technology | 1:1000 |
| Phospho-JNK (T183/Y185) | Cell Signaling Technology | 1:1000 |
| MAPK (Erk1/2) | Cell Signaling Technology | 1:1000 |
| Phospho-MAPK (Erk1/2) (T202/Y204) | Cell Signaling Technology | 1:1000 |
| p38 MAPK | Cell Signaling Technology | 1:1000 |
| Phospho-p38 MAPK (T180/Y182) | Cell Signaling Technology | 1:1000 |
| PEBP1 | ABclonal | 1:1000 |
| Phospho-PEBP1 (S153) | Abcam | 1:1000 |
| GAPDH | Proteintech | 1:5000 |

Table S3 His-FadA-precipitating cell proteins identified in PDLSCs.

| Accession code | Protein name | Protein description | MW (kDa) |
| --- | --- | --- | --- |
| P06703 | S100A6 | Protein S100-A6 | 10.2 |
| P0CG48 | UBC | Polyubiquitin-C | 77.0 |
| P07355 | ANXA2 | Annexin A2 | 38.6 |
| P26447 | S100A4 | Protein S100-A4 | 11.7 |
| P11142 | HSPA8 | Heat shock cognate 71 kDa protein | 70.9 |
| P30086 | PEBP1 | Phosphatidylethanolamine-binding protein 1 | 21.0 |
| P04083 | ANXA1 | Annexin A1 | 38.7 |
| O00299 | CLIC1 | Chloride intracellular channel protein 1 | 26.9 |
| P05362 | ICAM1 | Intercellular adhesion molecule 1 | 57.8 |

Table S4 Differentially expressed miRNAs at each time points of *F. nucleatum* infected PDLSCs

| Time points | Differentially expressed miRNA |
| --- | --- |
| 1h | hsa-miR-3118, hsa-miR-96-5p |
| 3h | hsa-miR-6747-5p, hsa-miR-5089-3p, hsa-miR-3940-5p,  hsa-miR-1181, hsa-miR-12127, hsa-miR-4804-5p,  hsa-miR-6853-3p, hsa-miR-6089 |
| 6h | hsa-miR-200a-5p, hsa-miR-4257, hsa-miR-4696,  hsa-miR-4472, hsa-miR-12127 |
| 12h | hsa-miR-4696, hsa-miR-4745-3p, hsa-miR-12127,  hsa-miR-3192-5p, hsa-miR-2278 |
